# Galvanic Displacement Synthesis of Monodisperse Janus‐ and Satellite‐Like Plasmonic–Magnetic Ag–Fe@Fe_3_O_4_ Heterostructures with Reduced Cytotoxicity

**DOI:** 10.1002/advs.201800271

**Published:** 2018-05-15

**Authors:** Huilin Zhang, Ziyu Yang, Yanmin Ju, Xin Chu, Ya Ding, Xiaoxiao Huang, Kai Zhu, Tianyu Tang, Xintai Su, Yanglong Hou

**Affiliations:** ^1^ Beijing Key Laboratory for Magnetoeletric Materials and Devices (BKL‐MEMD) Beijing Innovation Center for Engineering Science and Advanced Technology (BIC‐ESAT) Department of Materials Science and Engineering College of Engineering Peking University Beijing 100871 China; ^2^ College of Life Science Peking University Beijing 100871 China; ^3^ State Key Laboratory of Natural Medicines Department of Pharmaceutical Analysis China Pharmaceutical University Nanjing 210009 China; ^4^ Department of Chemistry School of Science Zhejiang Sci‐Tech University Hangzhou 310018 China

**Keywords:** Ag–Fe@Fe_3_O_4_, galvanic displacement, magnetic properties, reduced cytotoxicity

## Abstract

The unique physicochemical properties of silver nanoparticles offer a large potential for biomedical application, however, the serious biotoxicity restricts their usage. Herein, nanogalvanic couple Ag–Fe@Fe_3_O_4_ heterostructures (AFHs) are designed to prevent Ag^+^ release from the cathodic Ag by sacrificial anodic Fe, which can reduce the cytotoxicity of Ag. AFHs are synthesized with modified galvanic displacement strategy in nonaqueous solution. To eliminate the restriction of lattice mismatch between Fe and Ag, amorphous Fe@Fe_3_O_4_ nanoparticles (NPs) are selected as seeds, meanwhile, reductive Fe can reduce Ag precursor directly even at as low as 20 °C without additional reductant. The thickness of the Fe_3_O_4_ shell can influence the amorphous properties of AFHs, and a series of Janus‐ and satellite‐like AFHs are synthesized. A “cut‐off thickness” effect is proposed based on the abnormal phenomenon that with the increase of reaction temperature, the diameter of Ag in AFHs decreases. Because of the interphase interaction and the coupling effect of Ag and Fe@Fe_3_O_4_, the AFHs exhibit unique optical and magnetic properties. This strategy for synthesis of monodisperse heterostructures can be extended for other metals, such as Au and Cu.

## 1 Introduction

In over a decade, nanoscale heterostructures consisted of two or more functional nanoparticles (NPs) have attracted great interests due to the multifunctional properties not only from the superposition of each counterpart, but also the synergistic effect of the topological structure.^1^ The intimate contact between counterparts in the heterostructures should allow strong interactions and provide an efficient way for the modulation of the physical and chemical properties of the heterostructures.^2^ Among these properties, magnetic and optical properties can be efficiently controlled due to the synergistic effect and coupling effect.^3^ Especially, heterostructures consisted of noble metal and magnetic NPs, which could make good use of the special plasmonic resonance properties of noble metal counterpart and the unique magnetic properties of magnetic counterpart, have exhibited excellent potential applications in various fields such as multimodal imaging, targeting cancer therapy, and recyclable catalysis.^4^ Recently, plasmon‐enhanced two‐photon fluorescence (TPF) of Au and Ag NPs exhibit great advantages over conventional one‐photon fluorescent imaging.^5^ The complementary advantages of the magnetic resonance imaging (MRI) and TPF in one nanoprobe can endow probes the ability of accurate positioning of the overall tumor in the body and the fine local details, which is attractive for the early diagnosis of tumors.^6^ Compared with the widely studied Au‐based NPs, Ag‐based NPs are seldom used in biomedical field, except for antimicrobial, mainly due to the serious cytotoxicity of Ag^+^ released from Ag NPs.^7^ However, the unique properties of Ag NPs, such as special optical property, excellent electrical conductivity, good catalytic ability, bactericidal ability, and so on, are promising for biomedical application except their toxicity.^8^ Thus, efforts to reduce the biotoxicity of Ag are urgent needed. For bone implant, some researchers found that Ag NP–loaded Zn materials have obvious improved bactericidal ability but low cytotoxicity because of the microgalvanic couple formation that anode Zn can protect cathode Ag from being oxidized.^9^ Galvanic couples are efficient way to reduce the toxicity of cathodic Ag. Thus, the heterogeneous combination of Fe and Ag galvanic couples are hopeful to take full advantages of the unique properties of Ag and Fe, as well as reduce the cytotoxicity.

Until now, many efforts have been developed for the synthesis of plasmonic–magnetic heterostructures with Janus or core@shell structures,^10^ such as, dumbbell‐like Au–Fe_3_O_4_ NPs and Fe_3_O_4_@Au core@shell NPs.^11^ We recently reported the synthesis of Au–Fe_2_C Janus NPs for triple‐modal imaging–guided tumor photothermal therapy.^12^ Compared with the Au‐based heterostructures, the synthesis of Ag‐based heterostructures is suffering from the lower surfactivity, higher instability, and lower melting point of the Ag NPs, which is much more difficult for morphology controlling.^13^ To our knowledge, there are no reports of heterostructures combined of zerovalent Fe and Ag. Even though metal–magnetic heterostructures have been reported by thousands of papers, very few methods can synthesize monodisperse materials mainly due to the large lattice mismatch. Therefore, an easier method with simplified and repeatable approaches for the synthesis of monodisperse noble metal–magnetic heterostructures is quite needed.

Galvanic displacement is a well‐known method to prepare bimetallic heterostructures with core–shell, hollow, and nanocage structures.^14^ Nevertheless, the heterogeneous nucleation structures with exposed interface, like Janus‐like and satellite‐like heterostructures, are attractive for some specific studies which need specific surface and interface exposure. Herein, we report the synthesis of Janus‐ and satellite‐like Ag–Fe@Fe_3_O_4_ heterostructures, respectively, with modified galvanic displacement in nonaqueous solution. Considering that amorphous materials have been demonstrated as an efficient way to nonepitaxial growth heterostructures with large lattice mismatches,^15^ amorphous Fe@Fe_3_O_4_ NP was selected both as the reductant and as seeds, in which the zerovalent Fe has strong reduction activity, and the amorphous shell can not only protect the structure, but also eliminate the restriction of large lattice mismatch between Fe and Ag. Moreover, the synthesis of novel metal oleate complex as precursor, silver oleate (AgOA) as an example, allows the complete connection of AgOA and oleylamine‐coated Fe@Fe_3_O_4_ NPs in the nonaqueous solution to make sure that the reaction happened homogenously. Both Janus‐ and satellite‐like heterostructures with controlled Ag sizes can be synthesized by carefully tuning the reaction temperature or the shell thickness, resulting in the controlled optical and magnetic properties.

## 2 Results and Discussion

**Scheme 1:**
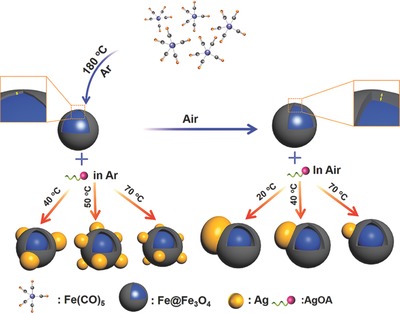
Schematic illustration of the synthetic process of the Janus‐ and satellite‐like Ag–Fe@Fe_3_O_4_ heterostructures.

The synthetic procedure of Ag–Fe@Fe_3_O_4_ heterostructures (AFHs) involves the initial synthesis of AgOA complex as precursors, the synthesis of amorphous Fe@Fe_3_O_4_ NPs as seeds, and the subsequent galvanic displacement between Fe@Fe_3_O_4_ and AgOA. As illustrated in **Scheme** **1**, the amorphous Fe@Fe_3_O_4_ NPs were prepared via the pyrolysis of iron pentacarbonyl (Fe(CO)_5_) in the 1‐octadecene (ODE) and oleylamine (OAm).^16^ When washed and exposed in air, the outermost Fe of as‐prepared NPs was oxidized, and a layer of iron oxide shell was formed, the resulted sample is referred as Fe@Fe_3_O_4_ NPs. However, the oleylamine‐coated Fe@Fe_3_O_4_ NP is hard to connect with the conventional oleophobic silver compound like silver nitrate (AgNO_3_). In order to solve this problem, the oleophilic AgOA was prepared through an easy but high‐efficiency two‐phase method, as illustrated in Figure S1 (Supporting Information). According to the periodic law, Ag can be recovered from its ionic states by displacing with active metal, such as Fe. When the fresh prepared AgOA meets the Fe@Fe_3_O_4_ in solution, the recovered Ag just attached on the amorphous surface of the Fe@Fe_3_O_4_ NPs, resulting in the Janus‐like Ag–Fe@Fe_3_O_4_ heterostructures (JAFHs). The size of Ag islands can be tuned depending on the reaction temperature. Note that if the inert atmosphere is introduced and kept throughout the reaction, another structure distinguished from Janus one, i.e., the satellite‐like Ag–Fe@Fe_3_O_4_ heterostructures (SAFHs) with several Ag NPs distributed on the surface of Fe@Fe_3_O_4_ NPs can be obtained. It is worth noting, in both of the cases, that the amorphous iron oxide shell plays a critical role for grafting of Ag NPs by avoiding the lattice mismatch restriction between Ag and Fe_3_O_4_. As, it is well known that the lattice mismatch is the most restriction factor for the fabrication of heterostructures.^17^ In order to identify the viewpoint, both amorphous and crystalline Fe@Fe_3_O_4_ NPs with similar sizes and morphologies were synthesized and characterized (Figure S2, Supporting Information) for the contrast experiments.

**Figure 1:**
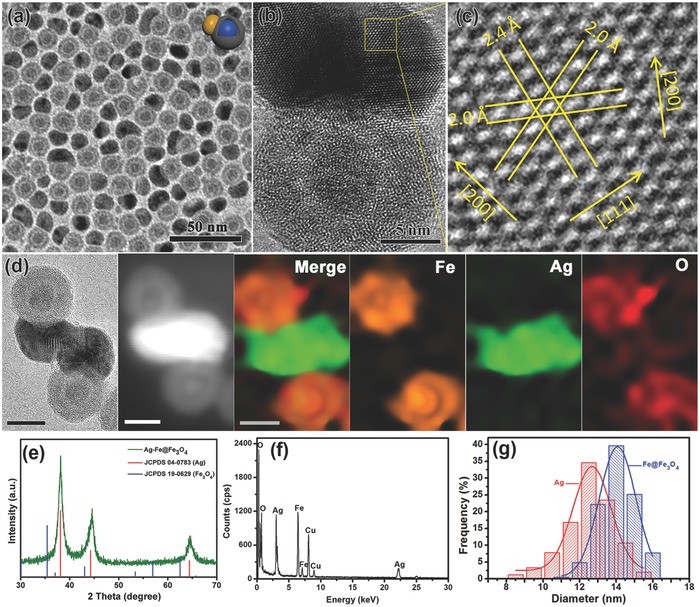
a) Low‐ and b,c) high‐resolution TEM images of JAFHs synthesized at room temperature (≈25 °C). d) The representative TEM image of JAFHs and the corresponding dark‐field high‐angle annular dark field scanning transmission electron microscopy (HAADF‐STEM) image and element mappings for Fe, Ag, and O. Scale bar in (d) = 10 nm. e) XRD patterns and f) EDS spectrum of JAFHs. g) The corresponding size distributions of Ag and Fe@Fe_3_O_4_ in JAFHs.

The typical morphology of JAFHs was characterized by transmission electron microscopy (TEM). As shown in **Figure** **1**a, the dark‐colored Ag decorated on the surface of Fe@Fe_3_O_4_ NPs. The obvious gap between Fe core and Fe_3_O_4_ shell could be observed, indicating that the reaction happened between Ag^+^ and the internal Fe, that the different diffusion rate between the Fe consumption and the Fe_3_O_4_ generation process generated the gap according to the Kirkendall effect.^18^ To reveal the exact structure of sample, high‐resolution transmission electron microscopy (HRTEM) was employed. As shown in Figure 1b, the crystalline Ag hat coated on the amorphous Fe@Fe_3_O_4_ NPs. The fringes with a separation of 2.4 and 2.0 Å corresponded to (111) and (200) reflection, respectively, which is allowed for face center cubic (fcc) lattice of Ag (Figure 1c). The energy dispersive spectroscopic (EDS) element mappings of Ag, Fe, and O further confirm that the Ag hats were firmly attached on the Fe@Fe_3_O_4_ NPs (Figure 1d). The EDS spectra of JAFHs are shown in Figure 1f. Figure 1e shows the X‐ray diffraction (XRD) pattern of JAFHs that only diffraction peaks of Ag were detected, the absence of the characteristic peaks related to Fe and Fe_3_O_4_ further confirms the fcc structure of Ag and the amorphous feature of Fe@Fe_3_O_4_ part. The statistic size distributions of Ag and Fe@Fe_3_O_4_ of the heterostructures are shown in Figure 1g, the average diameter of Ag and Fe@Fe_3_O_4_ are 12.7 and 14 nm, respectively. By contrast, the crystalline Fe@Fe_3_O_4_ NPs were used in the experiment under the same conditions, as shown in Figure S3 (Supporting Information), Ag was also produced but no heterostructures were formed due to the lattice mismatch. As the results described above, it can be concluded that the amorphous character of Fe@Fe_3_O_4_ NPs plays a critical role in the fabrication of heterostructures by eliminating the lattice mismatch.

In order to identify which constitute contributes to the Ag recovery, we synthesized hollow Fe_3_O_4_ NPs with similar size as seeds to react with the AgOA complex, however, no Ag obtained at the same conditions, as shown in Figure S4 (Supporting Information), indicating that the Fe_3_O_4_ shell cannot recover the Ag from AgOA. When AgOA mixed with OAm only in hexane or in ODE, there were also no Ag recovered even heated to 100 °C. It is generally accepted that the galvanic displacement could be happened when relatively active metal directly contact with the inert metal ion as the electron can transfer directly from the metal to the ion. However, in this study, we found that galvanic displacement also can happen even though the Fe and AgOA were divided by thin Fe_3_O_4_ shell. Actually, the Fe_3_O_4_ shell was naturally oxidized from Fe core, thus there may be natural transition of elementary composition from Fe to Fe_3_O_4_ in the interface with rich oxygen vacancy, and also generate the efficient electron density gradient, as illustrated in Figure S5 (Supporting Information). The thinner the shell is, the higher efficient electron density of the surface has, and easier for the galvanic displacement to happen, vice versa. As a result, the Fe@Fe_3_O_4_ as a whole, the efficient surface electron density will influence the displacement ability to the AgOA. However, when the shell thickness reached to the distance, the low efficient surface electron density will cut off the displacement. The pinhole on the oxide shell may also contribute to the electron transfer at the initial stage of the reduction.

**Figure 2:**
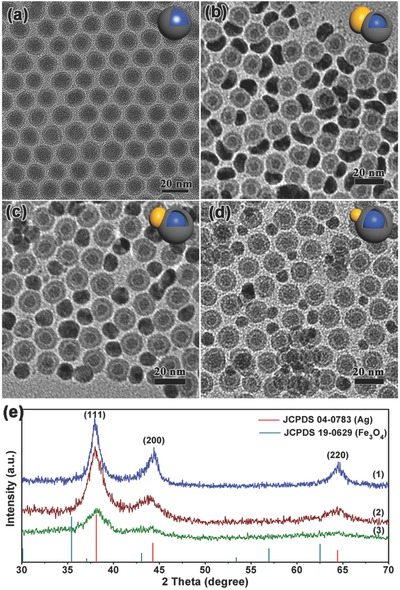
TEM images of a) amorphous Fe@Fe_3_O_4_ NPs and b–d) JAFHs with different‐sized Ag islands synthesized at relevant temperature: b) 15 nm at 20 °C, c) 10 nm at 40 °C, and d) 5 nm at 70 °C, insets highlight the representative individual particles with cover area mark. e) The corresponding XRD patterns of JAFHs.

Considering that the temperature could affect the reaction rate, the experiments were further carried out at 20, 40, and 70 °C, respectively, and the Fe@Fe_3_O_4_ NPs (**Figure** **2**a) from the same batch were used to eliminate extraneous influence. As shown in Figure 2b–d, Ag on JAFHs can be tuned to various sizes. Interestingly, with the increase of reaction temperature, the sizes of Ag domains were decreased, which is abnormal to our knowledge that with the increase of temperature, the redox reaction will be faster and the Ag should be bigger for equal reaction time. This phenomenon may due to the existence of Fe_3_O_4_ shell. When Fe@Fe_3_O_4_ seeds were exposed to the AgOA solution at different temperatures, the Fe@Fe_3_O_4_ could be oxidized by not only AgOA but also the oxygen dissolved in the solution. At a lower temperature, such as 20 °C, the oxidation from the oxygen was very slow, which provided more chance for the displacement of Ag until the shell reached to the thickness that the galvanic displacement cannot happen. The result is that about 15 nm length–squashed Ag covered more than a quarter of Fe@Fe_3_O_4_ surface (Figure 2b). At 40 °C, the oxidation from the oxygen would be increased, while less Ag could be recovered before the shell reached to the critical thickness, resulting in smaller‐sized Ag domain (Figure 2c). When the reaction temperature increased to 70 °C, the oxygen would contribute the main oxidation of Fe@Fe_3_O_4_ and the Ag had no chance to grow bigger before the shell reached to the critical thickness, as shown in Figure 2d. The XRD patterns also exhibit only diffraction peaks of Ag (Figure 2e) due to the good crystalline feature of Ag but amorphous characters of Fe@Fe_3_O_4_ NPs. With the increase of reaction temperature, the increase of the full width at half maximum (FWHM) in XRD patterns (Figure 2e and Table S1 (Supporting Information)) indicated the decrease of Ag sizes, which is in accord with the TEM information. As described above, it can be pointed out that the shell thickness should have great influence on the galvanic displacement between Fe and AgOA.

In order to examine the influence of shell, we further synthesized the Fe@Fe_3_O_4_ seeds with average shell thicknesses of 1.1, 2.2, and 3.1 nm, respectively (Figures S6a–c and S7a–c, Supporting Information), by carefully controlling the oxidation process. After mixing with AgOA at 40 °C for 3 h, SAFHs with several Ag NPs attached on each NP (Figure S6d, Supporting Information) were synthesized using the seeds with 1.1 nm shell, while JAFHs (Figure S6e, Supporting Information) were obtained in the case of the seeds with 2.2 nm shell. However, when the average shell thickness was enlarged to 3.1 nm, the silver NPs became smaller and about 68% of the Fe@Fe_3_O_4_ NPs without Ag dots (Figure S6f, Supporting Information). It is in good agreement with the shell thickness distributions (Figure S6c, Supporting Information) that more than 65% of Fe@Fe_3_O_4_ seeds had the shell thicker than 3 nm. These phenomena indicate that the Fe_3_O_4_ shell can inhibit the reaction by increasing the thickness and decreasing the efficient electron density on surface. The thicker of the shell is, the lower the efficient electron density has and the harder for the galvanic displacement to happen. It is easy to understand that there must be a critical thickness of the shell that determines whether galvanic displacement can happen. We define this shell thickness as “cut‐off thickness,” which means that the reaction would be cut off when the shell reaches to that thickness. The “cut‐off thickness” maybe different depending on the reactants, medium, and conditions. And, the “cut‐off thickness” in this reaction system may be about 3.0 nm. Regardless of the shell thickness of the Fe@Fe_3_O_4_ seeds, almost all particles had the shell thicker than 3.0 nm after the reaction (Figure S7, Supporting Information).

To understand the nucleation and growth mechanism, we monitored the synthetic progress at an interval of 30 min from the beginning of the reaction, and the TEM images are shown in Figure S8 (Supporting Information). When the reaction started for 10 min, a handful of small Ag NPs were detected on the Fe@Fe_3_O_4_ NPs (Figure S8a, Supporting Information). With the reaction going on, Ag islands were grown larger and more Fe@Fe_3_O_4_ NPs were decorated with Ag (Figure S8b–e, Supporting Information). After 150 min, most of Fe@Fe_3_O_4_ NPs were attached with Ag NPs (Figure S8f, Supporting Information), but not uniform, mainly because the nucleation did not start at the same time considering the various conditions of Fe@Fe_3_O_4_ seeds. After 180 min, Ag islands grew uniform (Figure S8g, Supporting Information), but did not grow larger even for a longer time (Figure S8h, Supporting Information).

**Scheme 2:**
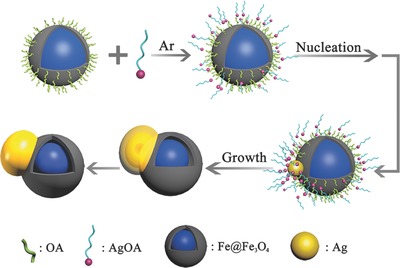
Schematic illustration of the nucleation and growth mechanism of the Ag–Fe@Fe_3_O_4_ heterostructures.

From the process mentioned above, the mechanism for the formation of AFHs through galvanic displacement can be summarized, as illustrated in **Scheme** **2**. The process includes the connection of AgOA and Fe@Fe_3_O_4_ NPs, the nucleation and growth of Ag. First, when dissolved in the low‐polar solvent, the hydrophobic AgOA molecules attached to the oleylamine‐coated Fe@Fe_3_O_4_ NPs by van der Waals' force. The displacement first happened on the relatively thinner site of the shell with higher efficient electron density. The irregular arrangement of the iron and oxygen atoms in amorphous iron oxide shell helps the Ag atoms root in the surface, as illustrated in Figure S9 (Supporting Information), however, the crystalline shell with large lattice mismatch would not allow the Ag attach on the surface. Once the Ag nucleated and rooted in the amorphous shell, the subsequently reduced Ag atoms would epitaxially grow around the Ag nucleus layer by layer. Meanwhile, the Fe core lost electron and was oxidized to be Fe_3_O_4_ and diffused to the shell, resulting in the gradually thicker Fe_3_O_4_ shell and the reduced Fe core. When the thickness of the Fe_3_O_4_ shell reached to the “cut‐off thickness,” the reaction would be stopped and the size of Ag would be fixed, resulting in the formation of AFHs.

**Figure 3:**
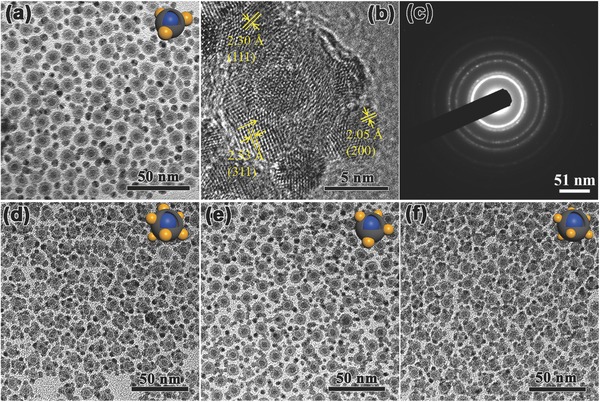
TEM images of SAFHs synthesized at various temperatures: a) 40 °C, d) 50 °C, e) 60 °C, and f) 70 °C. b) HRTEM image and c) SAED patterns of the representative SAFHs in (a).

Accordingly, if the shell can be controlled thinner, there may be multiposition for the Ag nucleation and growth in the displacement reaction. The key point is to avoid oxygen to get in the system. We made efforts to exclude the oxygen in the AgOA solution by vacuuming the solution with a vacuum pump and protecting it under argon (Ar) atmosphere, and the Fe@Fe_3_O_4_ seeds were rapidly used as synthesized in their original solution under Ar atmosphere to avoid oxygen. And, a degassed and Ar‐protected long needle syringe was used for the AgOA injection. As can be seen in **Figure** **3**, as expected, monodisperse SAFHs with a number of ultrasmall Ag NPs decorated around the Fe@Fe_3_O_4_ NPs were obtained. The HRTEM image exhibits the high crystalline Ag islands decorated on the amorphous part of the partly crystalline Fe_3_O_4_ shell as well as the clear interface, which is in good agreement with the phenomenon in the Janus‐like structures. The lattice spaces of 2.30 and 2.05 Å were corresponding to (111) and (200) planes of Ag, while the 2.53 Å is corresponding to the (311) plane of Fe_3_O_4_. The obvious widening diffraction peaks in XRD patterns (Figure S10, Supporting Information) revealed the small scale of Ag domain.

The reaction temperature also can influence the size of Ag in the satellite‐like structures. As shown in Figure 3d–f and Figure S11 (Supporting Information), the diameters of Ag domains decreased from 5.5 to 3.6 nm with increase of reaction temperature from 40 to 70 °C. This phenomenon is also in agreement with that in the Janus‐like structures. With increase of the reaction temperature, the reaction rate would be faster that may bring forward the “cut‐off thickness.”

**Figure 4:**
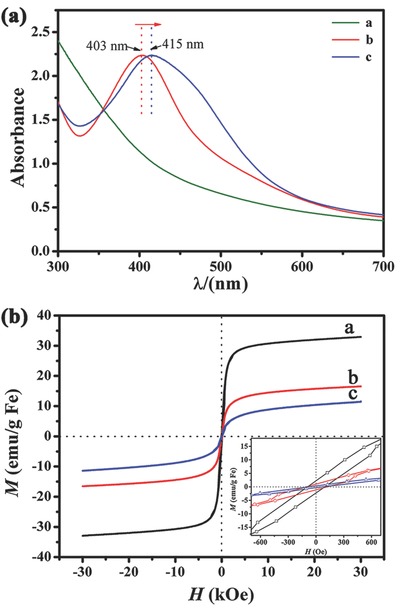
a) UV–vis absorption spectra and b) room temperature magnetic hysteresis loops of the samples (sample a: Fe@Fe_3_O_4_ NPs shown in Figure 2a, sample b: JAFHs shown in Figure 1a, sample c: SAFHs shown in Figure 3c). The inset in (b) highlight the area close to the origin.

The surface plasmon resonance (SPR) adsorption of Fe@Fe_3_O_4_, JAFHs (shown in Figure 1a), and SAFHs (shown in Figure 3c) were compared in **Figure** **4**a. Comparing with the Fe@Fe_3_O_4_ NPs, the AFHs showed strong absorption peaks in the visible region owing to the unique plasmonic adsorption of Ag. With the similar content of Ag (41.3% of JAFHs and 40.1% of SAFHs according to the inductively coupled plasma‐atomic emission spectrometer (ICP‐AES) measurement), the absorption maxima of the SAFHs shifted to longer wavelengths (415 nm), which may be due to the increase of symmetry of the satellite‐like structure. According to the plasmon mode coupling theory, the symmetric combination results in lower energy (redshift), while the antisymmetric combination results in higher energy (blueshift).[[qv: 3c,19]] In addition to the major peak, the SAFHs also exhibited a broad shoulder peak in the range of 450–500 nm that may be attributed to the near‐field coupling between the adjacent Ag islands on each heterostructure.^20^ Compared with the similar sized of pure Ag NPs (Figure S12, Supporting Information), the absorption peaks of both JAFHs and SAFHs exhibited distinct blueshifts (Figure S13a,b, Supporting Information). That can be explained by the variation of the efficient electron density on Ag. As reported, the excess electrons in the novel metal NPs can lead to a blueshift of the plasmonic absorption, while the electron deficiency will cause a redshift.[[qv: 3c,19]] The excess of electron on Ag may be supplied from the free electron on the Fe@Fe_3_O_4_ domain through the interface. Furthermore, the influence of Ag sizes to the adsorption also had been scrutinized. The absorption peak shifted to shorter wavelengths with the decrease of Ag sizes in JAFHs (Figure S13c, Supporting Information). Similar result was also detected in the SAFHs (Figure S13d, Supporting Information).The slight shift may contribute to the little changes of Ag diameters in the satellite‐like heterostructures. In turn, the obvious shift of the SPR absorption of the heterostructures also confirms the strong connection and interaction of the Ag and Fe@Fe_3_O_4_ NPs in the heterostructures.

In addition to the unique optical properties, the as‐prepared heterostructures also exhibited weak ferromagnetic properties owing to the Fe@Fe_3_O_4_ domain. The magnetic hysteresis loops of the pure Fe@Fe_3_O_4_, JAFHs, and SAFHs are compared in Figure 4b. The three samples showed the saturation magnetization (*M*_s_) of 32.9, 16.5, and 11.4 emu g^−1^, respectively, with the coercivity (*H*_c_) of 95, 120, and 60 Oe (inset in Figure 4b), respectively. The decrease of the *M*_s_ may attributed to the diamagnetic Ag in the heterostructures as well as the oxidation of the amorphous Fe core in the synthetic process.

**Figure 5:**
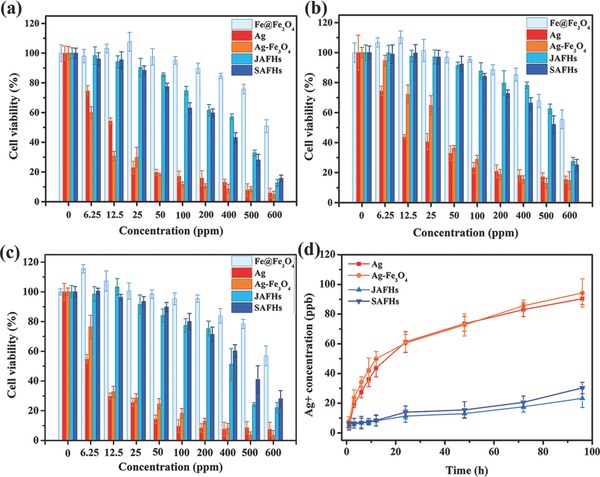
Cytotoxicity of JAFHs and SAFHs compared with Ag, Ag–Fe_3_O_4_, and Fe@Fe_3_O_4_ in different cell lines: a) 4T1, b) RAW264.7, and c) LO2. d) Ag^+^‐released concentration from the samples in PBS as a function of incubated time.

Owing to the combination of plasmonic and magnetic properties in the AFHs, they can be served as efficient TPF and MR dual‐modal imaging agents (as shown in **Figure** **5**). After the JAFHs being swallowed by 4T1 cancer cell, strong two‐photon fluorescence (red fluorescence in Figure 5c) was observed through excitation by femtosecond infrared laser pulses of 900 nm. The cell nucleus was stained with 4′,6‐diamidino‐2‐phenylindole (DAPI) and exhibited blue fluorescence (Figure 5b). The merging image implies that the JAFHs have been uptaken by 4T1 cell into the cytoplasm but not the nucleus (Figure 5d). The *T*_2_‐weighted MRI properties of JAFHs with different iron molar concentrations were evaluated at 3.0 T, as shown in Figure 5e, MRI signal intensity declines with the increasing of iron concentration. By calculation, the specific relaxivity value (*r*_2_) of JAFHs is estimated to be 128.3 mm ^−1^ s^−1^ (Figure 5f), which reveals the JAFHs have good potential as *T*_2_ contrast agents for diagnosis.

**Figure 6:**
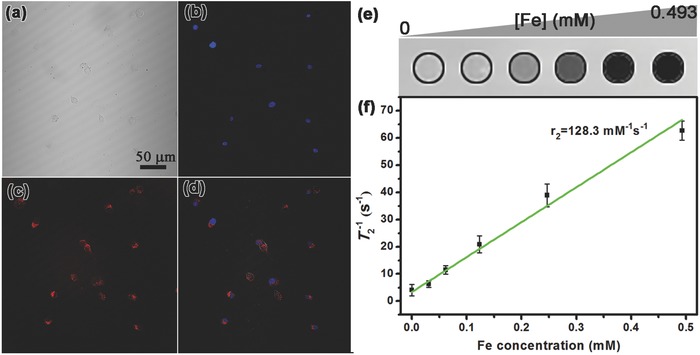
a) Bright‐field, b) one‐photon fluorescence of cell nucleus stained with DAPI, c) two‐photon fluorescence, and d) their overlay images of the 4T1 cells uptake JAFHs. e) *T*_2_‐weighted MR images and f) the corresponding *T*_2_ relaxation rates of JAFHs as a function of Fe concentration ([Fe] (× 10^−3^ m)).

In order to test the cytotoxicity of the synthesized samples, 4T1 mouse breast tumor cell line, RAW264.7 mouse macrophage cell line and LO2 normal human liver cell line were selected for 3‐[4,5‐dimethylthiazol‐2‐yl‐]‐2,5‐diphenyltetrazolium (MTT) experiment. As shown in **Figure** **6**, both JAFHs and SAFHs exhibited much lower cytotoxicity in three different kinds of cell lines compared with pure Ag and Ag–Fe_3_O_4_ NPs at same Ag concentration. Compared with the similar‐sized and ‐structured Ag–Fe_3_O_4_ NPs (Figure S14, Supporting Information), zerovalent Fe and Ag in JAFHs and SAFHs in cytoplasm constitute galvanic couples, the accelerated oxidation anodic Fe can protect the cathodic Ag from being oxidized, which will reduce the Ag^+^ release from JAFHs and SAFHs, so as to reduce the cytotoxicity. This is also being proved by the Ag^+^ release detection in phosphate buffer saline (PBS) by inductively coupled plasma mass spectrometry (ICP‐MS). As shown in Figure 6d, both JAFHs and SAFHs showed less Ag^+^ release compared with pure Ag and Ag–Fe_3_O_4_ NPs, this is consistent with the MTT results.

To examine the general applicability of the method reported here, the more active gold oleate complex (Au(OA)_3_) as well as much less active copper oleate complex (Cu(OA)_2_), compared with AgOA, were selected for the experiment. Au–Fe@Fe_3_O_4_ heterostructures were easily obtained at room temperature (Figure S15, Supporting Information), while Cu–Fe@Fe_3_O_4_ heterostructures can hardly be generated until the temperature increased to 120 °C for 5 h (Figure S16a–c, Supporting Information). However, the pure Cu(OA)_2_ cannot be decomposed by oleylamine at the same condition (Figure S16d, Supporting Information), indicating that the Cu was recovered by Fe@Fe_3_O_4_.

## 3 Conclusions

In summary, a facile‐modified galvanic displacement method was generated to synthesize monodisperse Janus‐ and satellite‐like Ag–Fe@Fe_3_O_4_ plasmonic–magnetic heterostructures using amorphous Fe@Fe_3_O_4_ NPs as seeds as well as reductive agent. Under this strategy, the replacement could be carried out at as low as 20 °C without additional reductant. The “cut‐off thickness” effect was proposed based on the abnormal phenomenon that with the increase of the reaction temperature, the sizes of Ag domain in heterostructures were decreased. Under the guidance of “cut‐off thickness” effect, a family of uniform Janus‐ and satellite‐like AFHs with tunable Ag sizes were obtained. Owing to the strong interphase interaction and the coupling effect of Ag and Fe@Fe_3_O_4_, the heterostructures exhibited tunable optical properties and favorable magnetic properties, which were further reflected in the good performance of plasmon‐enhanced TPF and MR dual‐modal imaging. The galvanic couples of Ag and Fe effectively reduce the cytotoxicity of JAFHs and SAFHs by reducing Ag^+^ release from cathodic Ag, which would open the door for Ag‐based application in biomedical studies.

## 4 Experimental Section

*Materials*: AgNO_3_ (≥99.9%), ODE (90%), ammonium bromide (NH_4_Br, ≥99.0%), borane‐*tert*‐butylamine complex (TBAB, 97%), and 4‐nitrobenzoic acid (4‐NP, 99%) were purchased from Alfa Aesar. OAm (70%), iron pentacarbonyl (Fe(CO)_5_, 99.9%), and dimethyl sulfoxide (DMSO) were purchased from Sigma‐Aldrich. Gold chloride trihydrate (HAuCl_4_·3H_2_O, ≥99.9% trace metals basis) was purchased from STREM chemicals. Sodium oleate (NaOA, >97%) was purchased from TCI. Deionized water (DI water, 18.2 MΩ) was refined from a Milli‐Q Ultrapure Water System. Other solvents including normal hexane (*n*‐hexane, ACS grade), ethanol (AR grade), and chloroform (CHCl_3_, ≥99.9%) were purchased from Beijing Chemical Works. All chemicals were directly used without further purification.

*Measurements*: XRD patterns were obtained using a Rigaku DMAX‐2400 X‐ray diffractometer with Cu Kα radiation (λ = 1.5405 Å). TEM images were taken on an FEI Tecnai T20 microscope with an acceleration voltage of 200 kV, and HRTEM images were obtained on an FEI Tecnai G20 F30 microscope and operated on an acceleration voltage of 300 kV. UV–visible spectra were measured using a UV‐2550 Shimadzu UV–vis spectrophotometer. Magnetic study was carried out on a Quantum Design PPMS XL with fields up to 30 kOe at room temperature. The concentrations of Ag and Fe in the samples were quantified using an ICP‐AES (Profile, Leeman, USA).

*Methods—Synthesis of Metal Oleate or Oleylamine (MOA or MOAm, M = Ag, Au, Cu) Precursor*: Typically, 0.25 mmol of AgNO_3_ was dissolved in the mixture of DI water (10 mL) and ethanol (20 mL), followed by adding hexane (30 mL) to form a two‐phase solution (Figure S1a, Supporting Information). 3 mL fresh NaOA (0.1 m) aqueous solution was added dropwise under vigorous stirring for 30 min. Then, the mixture was stood for the stratification, the AgOA was dissolved in upper layer with white color (Figure S1b, Supporting Information). Finally, the AgOA solution was separated and washed with DI water for two times in a separating funnel (Figure S1c, Supporting Information) for further use. Copper oleate precursor (Cu(OA)_2_) was synthesized with Cu(NO_3_)_2_ instead of AgNO_3_ in the above process. The green color upper solution indicated the generation of Cu(OA)_2_. Gold oleylamine precursor (Au(OAm)_3_) was obtained with the similar approach using HAuCl_4_·3H_2_O and OAm instead of AgNO_3_ and NaOA. The molar rate of Au^3+^ and OAm was 1:3, and saffron yellow upper solution indicated the formation of Au(OAm)_3_.

*Methods—Synthesis of Fe@Fe_3_O_4_ NPs*: The amorphous Fe@Fe_3_O_4_ NPs (A‐FeNPs) were synthesized by the modified method reported by Peng et al.[[qv: 16a]] Briefly, in a four‐necked flask, ODE (20 mL) and OAm (0.5 mL) were mixed under vigorous magnetic stirring and degassed with a vacuum pump at 100 °C for 1 h and then ventilated argon (Ar). As soon as the mixture was heated to 180 °C, iron pentacarbonyl (Fe(CO)_5_, 0.7 mL) was quickly injected into the flask. After 30 min, the products were cooled down and washed with hexane and ethanol for three times and finally redispersed and stored in hexane. The Fe@Fe_3_O_4_ nanocrystals (FeNCs) were synthesized with the similar progress, except NH_4_Br (8 mg) added at the beginning.[[qv: 16b]] In order to examine the influence of the thickness of iron oxide shell to the JAFHs, the as‐prepared A‐Fe was exposed to air and heated at 80 °C for 0, 6, and 12 h, resulting in the average shell thickness of about 1.1, 2.2, and 3.1 nm, respectively. The hollow Fe_3_O_4_ NPs (h‐Fe_3_O_4_) were synthesized by heating the Fe@Fe_3_O_4_ NPs at 100 °C for 4 h and then heated to 250 °C for 10 h.

*Methods—Synthesis of JAFHs*: Typically, AgOA solution (20 mL, 15 × 10^−3^ m in hexane) was added in a four‐necked flask with a magnetic stir bar. After 20 min stirring under Ar at room temperature, 2 mL of amorphous Fe@Fe_3_O_4_ NPs (0.25 m) was injected. The color of the mixture was changed to dark brown gradually, indicating the generation of Ag. Three hours later, the products were collected and washed for three times and finally redispersed in 10 mL hexane and protected under Ar atmosphere.

*Methods—Synthesis of SAFHs*: First, ODE (5 mL) was mixed with the as‐prepared AgOA solution (20 mL, 12.5 × 10^−3^ m in hexane) and then degassed under Ar at 30 °C for 30 min in a four‐necked flask. Next, Fe(CO)_5_ (100 µL) was quickly injected into the adequately degassed and Ar‐protected solution (15 mL ODE and 0.5 mL OAm) at 180 °C and stirred for 30 min. As soon as the mixture was cooled down to 40 °C, the Ar‐protected AgOA solution was quickly injected into the as‐prepared amorphous Fe@Fe_3_O_4_ solution under Ar and reacted for 3 h.

*Methods—Ag^+^ Release Measurement*: The Ag^+^ release from the samples was monitored by ICP‐AES. 1 mL of samples at Ag concentration of 2 mg mL^−1^ was sealed into a dialysis bag with the cutoff molecular weight of 3000 Da. These dialysis bags were immersed into 40 mL PBS (pH = 7.0) in a shaking incubator at 37 °C with a shaking speed of 150 rpm. At given intervals, 1 mL of the solution was collected for Ag concentration (µg mL^−1^ (ppb)) analysis in ICP‐AES. And then, 1 mL of fresh PBS was added.

*Methods—Cell Culture*: All the cell lines were purchased from Shanghai Institute of Cells, Chinese Academy of Science. 4T1 mouse breast tumor cell line and LO2 normal human liver cell line were cultured in Roswell Park Memorial Institute (RPMI) 1640 (Gibco, USA) supplemented with 10% heat‐inactivated fetal bovine serum (FBS), 1% streptomycin/penicillin at 37 °C in humidified atmosphere with 5% CO_2_. RAW264.7 mouse macrophage cell line was cultured in the same condition in high‐glucose Dulbecco's modified Eagle medium (DMEM) (Gibco, USA).

*Methods—Cytotoxicity Measurement*: 4T1 mouse breast tumor cell line, RAW264.7 mouse macrophage cell line, and LO2 normal human liver cell line were seeded in 96‐well plates (5000 cells in 100 µL of corresponding culture medium per well). After incubated for 24 h, the mediums were replaced with fresh corresponding culture medium containing A‐Fe, Ag, Ag–Fe_3_O_4_, JAFHs, or SAFHs at Fe concentration (for A‐Fe) or Ag concentration (for Ag, Ag–Fe_3_O_4_, JAFHs, and SAFHs) of 6.25, 12.5, 25, 50, 100, 200, 400, 500, and 600 µg mL^−1^, respectively. After incubated for another 24 h, the cells were mildly washed with PBS for three times, then coincubated with MTT solution (0.5 mg mL^−1^ in 100 µL of PBS) at 37 °C for 4 h. Then, the MTT solution was replaced by 150 µL of DMSO, and the absorbance was tested on a microplate reader (Bio‐TekELx800, USA) at the wavelength of 490 nm.

## Conflict of Interest

The authors declare no conflict of interest.

## Supporting information

Supplementary
