# Supplementary material for: Galvanic Displacement Synthesis of Monodisperse Janus‐ and Satellite‐Like Plasmonic–Magnetic Ag–Fe@Fe3O4 Heterostructures with Reduced Cytotoxicity
Source: Adv Sci (Weinh). 2018 May 15;5(8):1800271. doi: 10.1002/advs.201800271 (PMC6096995; doi:10.1002/advs.201800271)
Supplement: Supplementary file 1 — Supplementary [file ADVS-5-1800271-s001.pdf]

Copyright WILEY-VCH Verlag GmbH & Co. KGaA, 69469 Weinheim, Germany, 2018.

Supporting Information

for *Adv. Sci.,* DOI: 10.1002/advs.201800271

Galvanic Displacement Synthesis of Monodisperse Janus- and Satellite-Like Plasmonic–Magnetic Ag–Fe@Fe_3O_4 Heterostructures with Reduced Cytotoxicity

Huilin Zhang, Ziyu Yang, Yanmin Ju, Xin Chu, Ya Ding, Xiaoxiao Huang, Kai Zhu, Tianyu Tang, Xintai Su, and Yanglong Hou**

Supporting Information

Galvanic Displacement Synthesis of monodisperse Janus- and Satellite-like Plasmonic-Magnetic Ag-Fe@Fe_3O_4 Heterostructures with Reduced Cytotoxicity

Huilin Zhang, Ziyu Yang, Yanmin Ju, Xin Chu, Ya Ding, Xiaoxiao Huang, Kai Zhu, Tianyu Tang, Xintai Su* and Yanglong Hou *

Dr. H. L. Zhang, Dr. Z. Y. Yang, Dr. Y. M. Ju, Dr. X. Chu, Dr. X. X. Huang, Dr. K. Zhu, Dr. T. Y. Tang, Prof. Y. Hou
Beijing Key Laboratory for Magnetoeletric Materials and Devices (BKL-MEMD), Beijing Innovation Center for Engineering Science and Advanced Technology (BIC-ESAT), Department of Materials Science and Engineering, College of Engineering, Peking University, Beijing 100871, China
E-mail: hou@pku.edu.cn
Prof. X. T. Su
Department of Chemistry, School of Science, Zhejiang Sci-Tech University, Hangzhou 310018, China
E-mail: suxintai827@163.com
Prof. Y. Ding
State Key Laboratory of Natural Medicines, Department of Pharmaceutical Analysis, China Pharmaceutical University, China.
Dr. Y. M. Ju
College of Life Science, Peking University, Beijing 100871, China

Ⅰ. Supporting methods

1.1 Synthesis of Ag NPs: 5 nm Ag NPs were synthesized by thermolysis of AgOA (0.5 mmol) in OAm (20 mL) at 180 °C for 10 min under Ar, while 10 nm Ag NPs were synthesized by slowly heating the mixture up to 280 °C under Ar and kept at this temperature for 1 h.

1.2 Synthesis of Cu-Fe@Fe_3O_4 heterostructures: The Cu-Fe@Fe_3O_4 heterostructures were synthesized by injecting the green $Cu(OA)_2$ solution to the hot Fe@Fe_3O_4 solution at 120°C under Ar and kept for 5 h before cooling down to room temperature.

1.3 Synthesis of Au-Fe@Fe_3O_4 heterostructures: The Au-Fe@Fe_3O_4 heterostructures consisting of 6 nm Au islands and 14 nm Fe@Fe_3O_4 domains were synthesized by substituting the AgOA solution with $Au(OAm)_3$ solution. Other

processes were similar to the synthesis of AFHs. The reaction was carried out at 20 °C for 3 h.

1.4 Synthesis of Ag-Fe_3O_4 heterostructures: 10 nm Ag NPs (~20 mg) dissolved in 10 mL ODE, 5 mL OA and 0.2 mL OAm solution then degassed under Ar at 70 °C for 30 min in a four-necked flask. The mixture was heated to 150 °C, and 0.15 mL of $Fe(CO)_5$ were injected into the solution and heated to 300 °C. After 1 h, the products were collected and washed for 3 times.

1.5 Phase transfer and biofunctionalization: mPEG-COOH (MW: 2000) was used to modify the synthesized heterostructures. Typically, synthesized nanoparticles (20 mg) were dispersed into dichloromethane (15 mL), followed by adding in mPEG-COOH (20 mg), after stirring for 10 h, ethanol (20 mL) was added to collect the NPs by centrifuging (15000 rpm for 10 min). The NPs were washed with ethanol and water once, and finally dissolved in DI water through ultrasonic dispersion for further use.

1.6 MR imaging measurement: The MR imaging measurements of JAFHs in water solution were performed on a 3.0T clinical MRI scanner (GE Signa 3.0 T). JAFHs at given Fe concentrations of 0, 0.0308, 0.0616, 0.1232, 0.2465, 0.4929 mM were dispersed in 1mL of DI water (18.2 MΩ) in 1.5 mL centrifuge tubes and placed into the MRI scanner. T_2 weighted fast spin-echo (FR-FSE) sequence with parameters of TR=3000 ms, TE=102 ms, slice thickness=3.0 mm was used and the images were analyzed at the workstation provided by GE heathcare.

1.7 Two-photon fluorescence microscopy measurement: To evaluate the TPF imaging properties of JAFHs, the 4T1 cells were seeded into a 12 mm-diameter glass bottom culture dish (2×10^4 cells/well) and allowed to grow for 24 h at 37 °C under 5% CO_2. JAFHs with a concentration of 10 ppm w ere added and co- incubated with 4T1 cells for another 4 h, then the cells were washed with PBS to remove the free JAFHs. 4’-6-diamidino-2-phenylindole (DAPI) was further used for cell nuclei staining. The TPF imaging of cells was performed using an Olympus FV1000 laser scanning multiphoton microscope with a Ti:Sapphire laser as the excitation source.

Ⅱ. Supporting figures and discussions

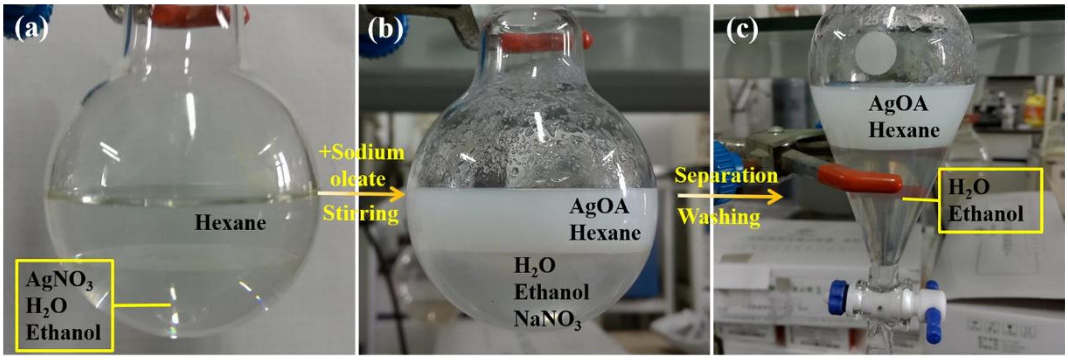

Figure S1. Photographs illustrate the synthesis of AgOA in a two-phase solution. The two-phase solution consists of a nonpolar solvent (n-hexane) in the upper layer and a polar solvent (mixture of H_2O and ethanol) in the under layer.

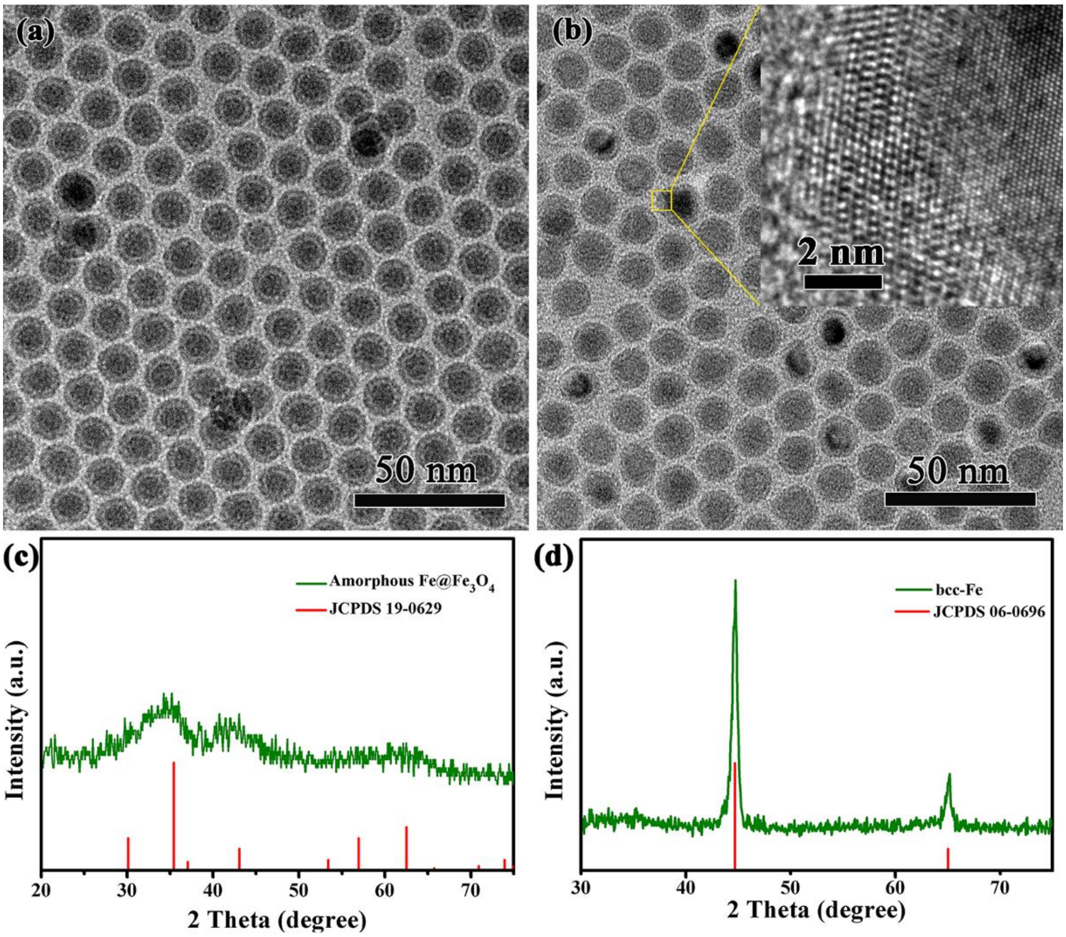

Figure S2. TEM images and XRD patterns of monodisperse A-FeNPs (a and c) and FeNCs (b and d), the inset in (b) shows the partial enlarged HRTEM view of the part of FeNCs.

Only unsharp Fe_3O_4 peaks appears in the XRD patterns in Figure S2c indicating the amorphous character of Fe@Fe_3O_4 in Figuree S2a. By comparison, when a small amount of NH_4Br added in the synthesis process, similar sized crystal Fe@Fe_3O_4 NPs were obtained (Figure S2b). From the XRD patterns in Figure S2d, the sharp peaks indicate the highly crystalline character of Fe core of the crystal Fe@Fe_3O_4 NPs, which is also proved by HRTEM image inserted in the Figure S2b, from which we can also see the crystalline character of the Fe_3O_4 shell.

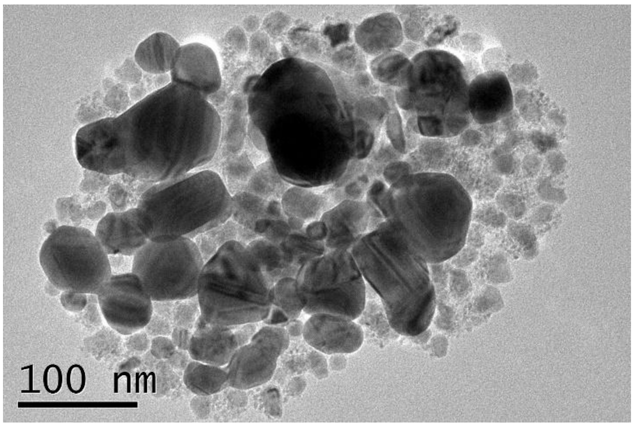

Figure S3. TEM image of products synthesized with crystalline Fe@Fe_3O_4 NPs as seeds and mixed with AgOA at 40 ^{o}C for 3 h. Even though the Ag can be reduced, but the lattice mismatch restraints the connection of Ag and Fe@Fe_3O_4.

Table S1. XRD patterns information of JAFHs in Figure 2e.

Sample	hkl	FWHM [a)]	Calculated crystalline (nm)	Average crystalline (nm) and standard deviation	TEM statistic average diameters (nm)
Ag in Figure 2e(1)	(111)	0.999	8.4	9.13±1.27	15
	(200)	0.816	10.6		
	(220)	1.120	8.4		
Ag in Figure 2e(2)	(111)	1.484	5.7	6.15±0.64	10
	(200)	1.401	6.6		
Ag in Figure 2e(3)	(111)	3.872	2.2	2.2	5

[a)](full width at half maximum (FWHM))

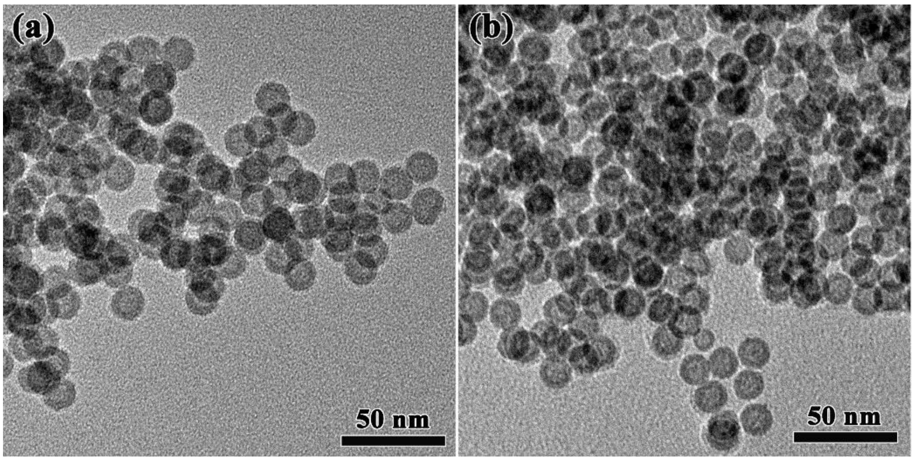

Figure S4. TEM images of (a) hollow Fe_3O_4 NPs and (b) NPs prepared with hollow Fe_3O_4 as seeds and mixed with AgOA at 40 ^oC for 3 h. There was no obvious changes and no Ag NPs appeared in the TEM images. That indicates Ag cannot be reduced by Fe_3O_4 at the same conditions.

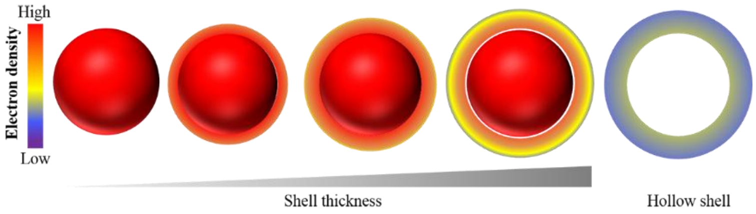

Figure S5. The demonstration of the efficient electron density distribution in Fe@Fe_3O_4 NPs based on the shell thickness.

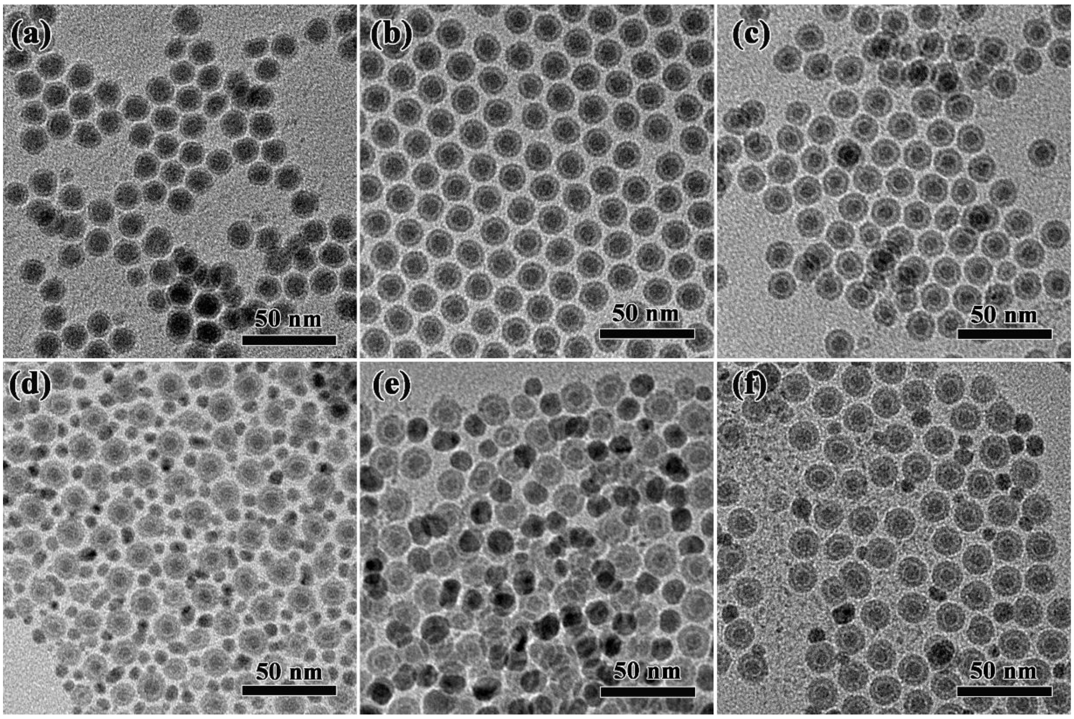

Figure S6. TEM images of $Fe@Fe_3O_4$ NPs with controlled oxidation in air at 80 ^oC for different with different iron oxide shell thickness (a) 0 h with1.1 nm shell, (b) 6 h with 2.2 nm shell and (c) 12 h with 3.1 nm shell, respectively, and (d-f) AFHs synthesized at 40 ^oC for 3 h with different morphology corresponding to Fe@ Fe_3O_4 seeds in (a-c).

$Fe@Fe_3O_4$ NPs with 1.1 nm iron oxide shell result in SAFHs (Figure S6d), with 2.2 nm shell result in JAFHs (Figure S6e). When the average iron oxide shell thickness rose to 3.1nm, the silver island was smaller and near half of the $Fe@Fe_3O_4$ NPs without Ag attachment (Figure S6f). When the thickness rose to a distance, the reaction can hardly happened. With thin shell, the silver nucleated and grew on multi sites of $Fe@Fe_3O_4$ seeds, resulting in the SAFHs. With a bit thicker shell, the surface efficient electron density would be decreased, and the silver nucleated and grew on the relatively thinner site of the shell, resulting in the JAFHs.

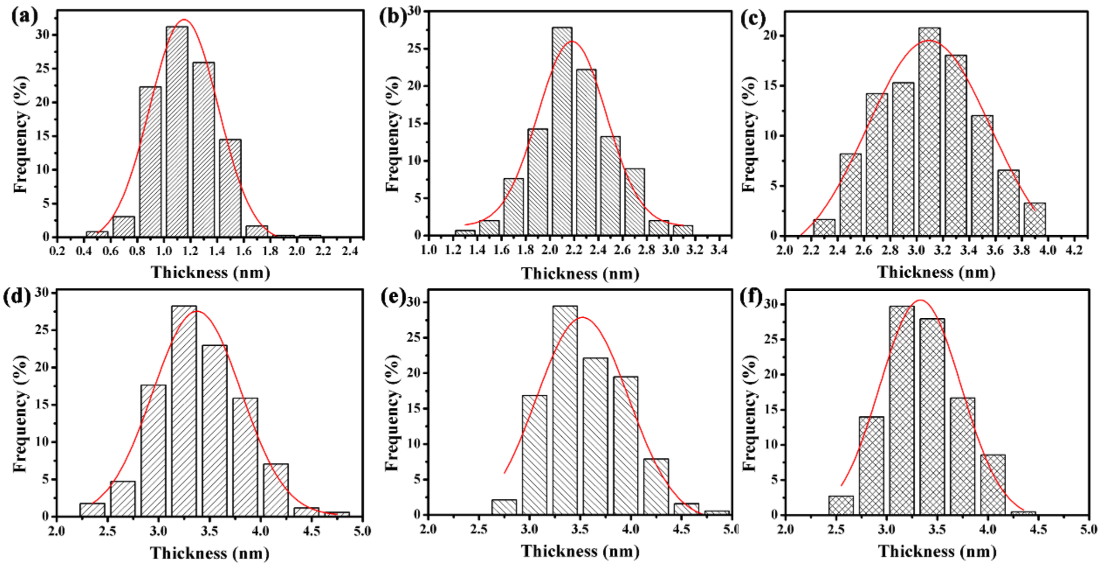

Figure S7. The iron oxide shell thickness distributions of Fe@Fe_3O_4 seeds oxidized in air at 80 ^{o}C for 0 h with an average thickness of 1.1 nm (a), 6 h with an average thickness of 2.2 nm (b) and 12 h with an average thickness of 3.1 nm (c), which is corresponded to Figure S6 (a-c); and (d-f) shows the iron oxide shell thickness distributions of the AFHs corresponded to Figure S6 (d-f), respectively.

The iron oxide shell thickness distributions of Fe@Fe_3O_4 seeds and Ag-Fe@ Fe_3O_4 NPs in Figure S6 are shown in Figure S7. For seeds, when oxidized for 1 h, the oxide shell thickness are mainly distributed between 0.9 nm to 1.5 nm with an average size of 1.1 nm. For 6 h oxidation, most of the iron oxide shell are distributed between 1.7 nm to 2.7 nm with an average size of 2.2 nm. Less than 5 % Fe@Fe_3O_4 NPs with shell thickness larger than 3.0 nm, which is the reason that a handful of single Fe@Fe_3O_4 NPs without Ag attaching in TEM images (Figure S6e). In Figure S6c, the majority of nanoparticles have shell thickness between 2.5nm to 3.7 nm with an average size of 3.1 nm. And approximately 65% of Fe@Fe_3O_4 NPs with shell thickness larger than 3.0 nm, which is agreement to the result in Figure S6f that about 68% of the single Fe@Fe_3O_4 NPs without Ag domain. Figure S7d-f show the iron oxide shell thickness after growing of Ag domain. It can be clearly seen that most of the NPs have shell thickness larger than 3.0 nm including the single Fe@Fe_3O_4, no matter what the sizes and numbers of Ag domains.

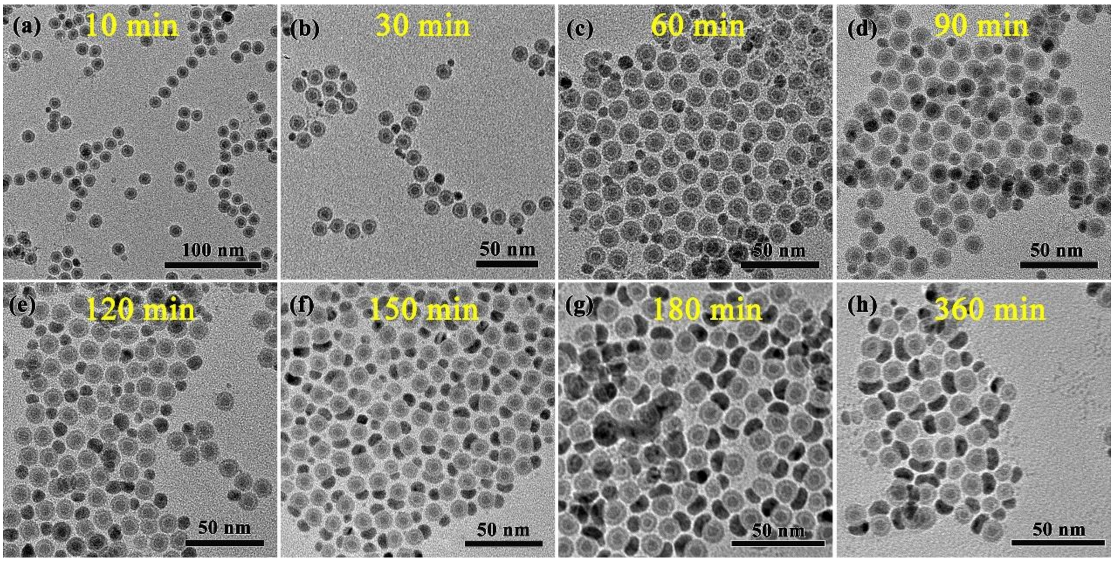

Figure S8. TEM images of Ag nucleation and growth process on Fe@Fe_3O_4 seeds reacted at 20 ^oC for (a) 10 min, (b) 30 min, (c) 60 min, (d) 90 min, (e) 120 min, (f) 150 min, (g) 180 min and (h) 360 min.

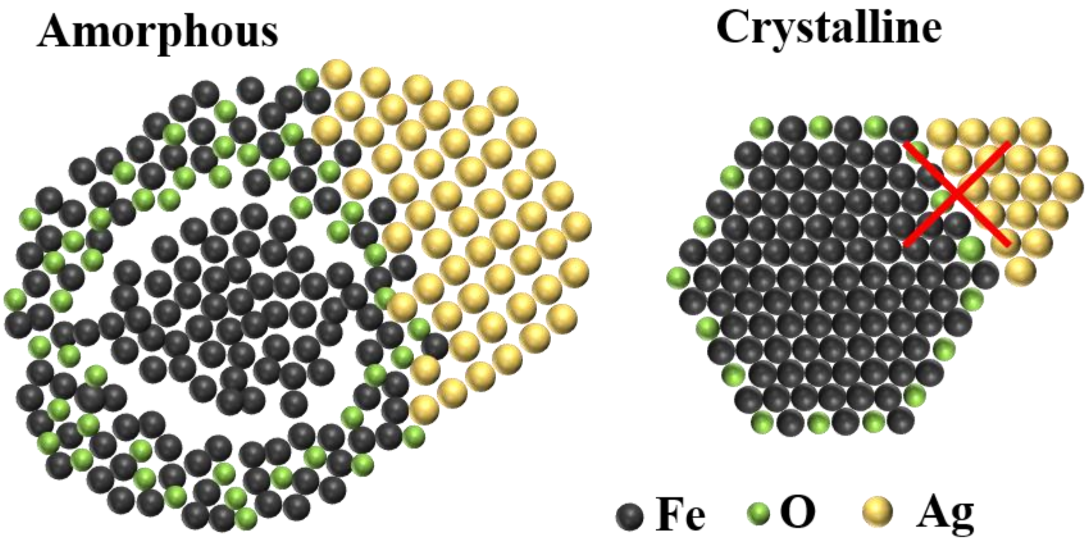

Figure S9. Scheme illustrate the mechanism of Ag atoms attach on amorphous iron oxide shell based on the irregular atoms spaces in the amorphous shell, but cannot attach to the crystalline iron oxide shell due to the lattice mismatch.

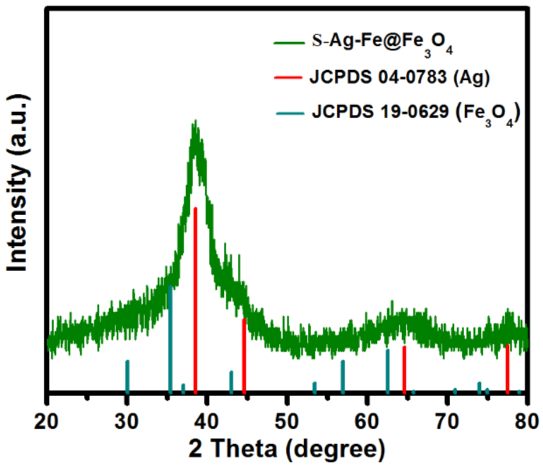

Figure S10. XRD patterns of SAFHs.

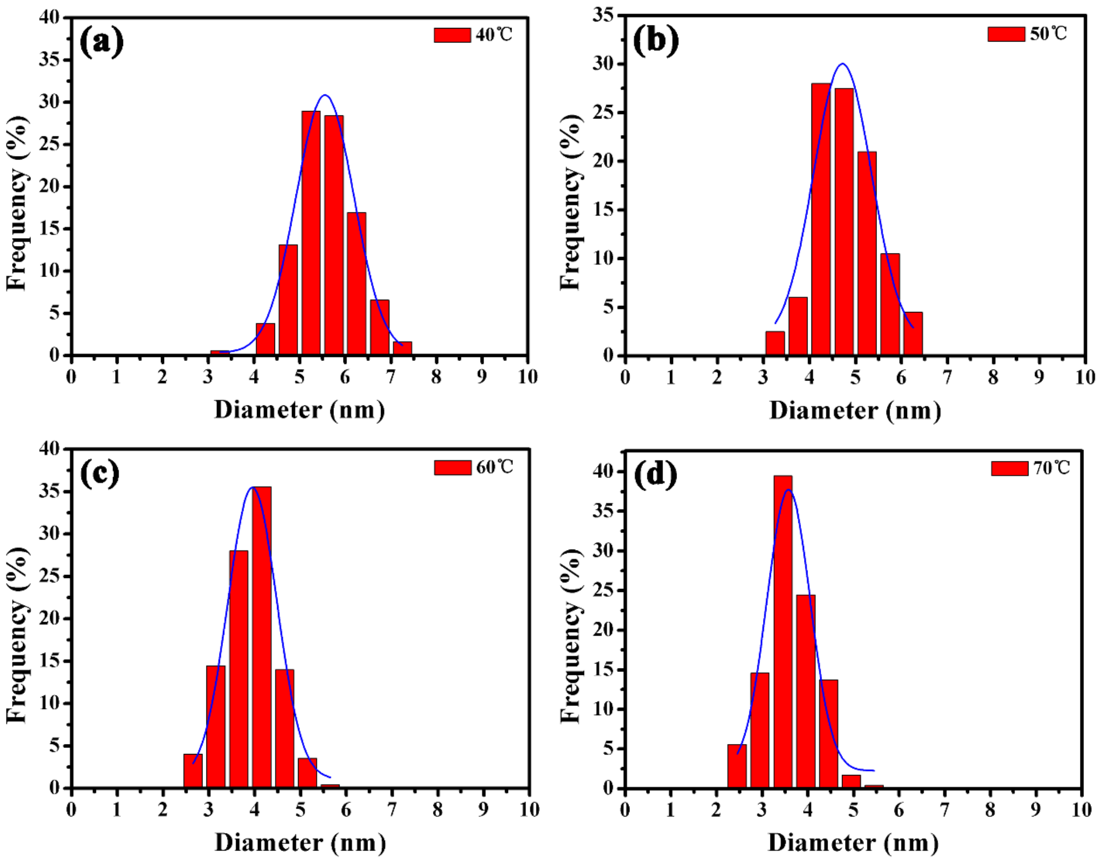

Figure S11. Size distribution of Ag domain in SAFHs synthesized at different temperatures as shown in Figure 4. The average diameter of the Ag domains is (a) 5.5 nm, (b) 4.7 nm, (c) 3.9 nm and (d) 3.6 nm, respectively.

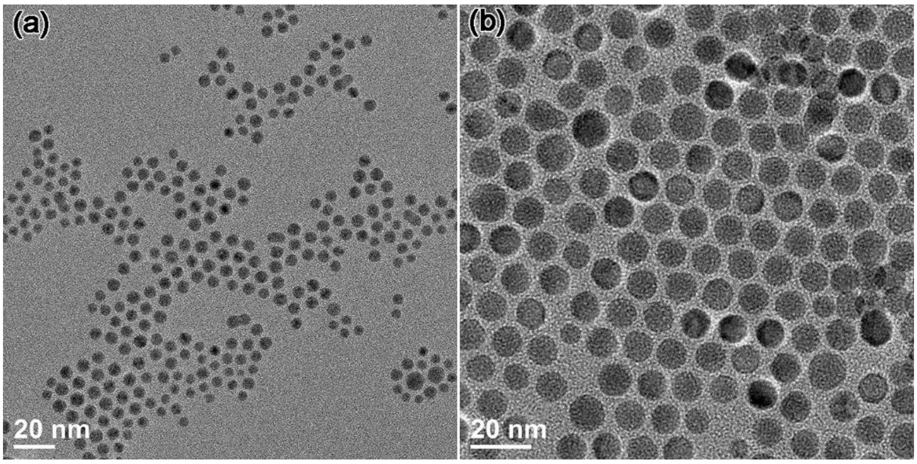

Figure S12. TEM images of (a) 5 nm Ag NPs and (b) 10 nm Ag NPs.

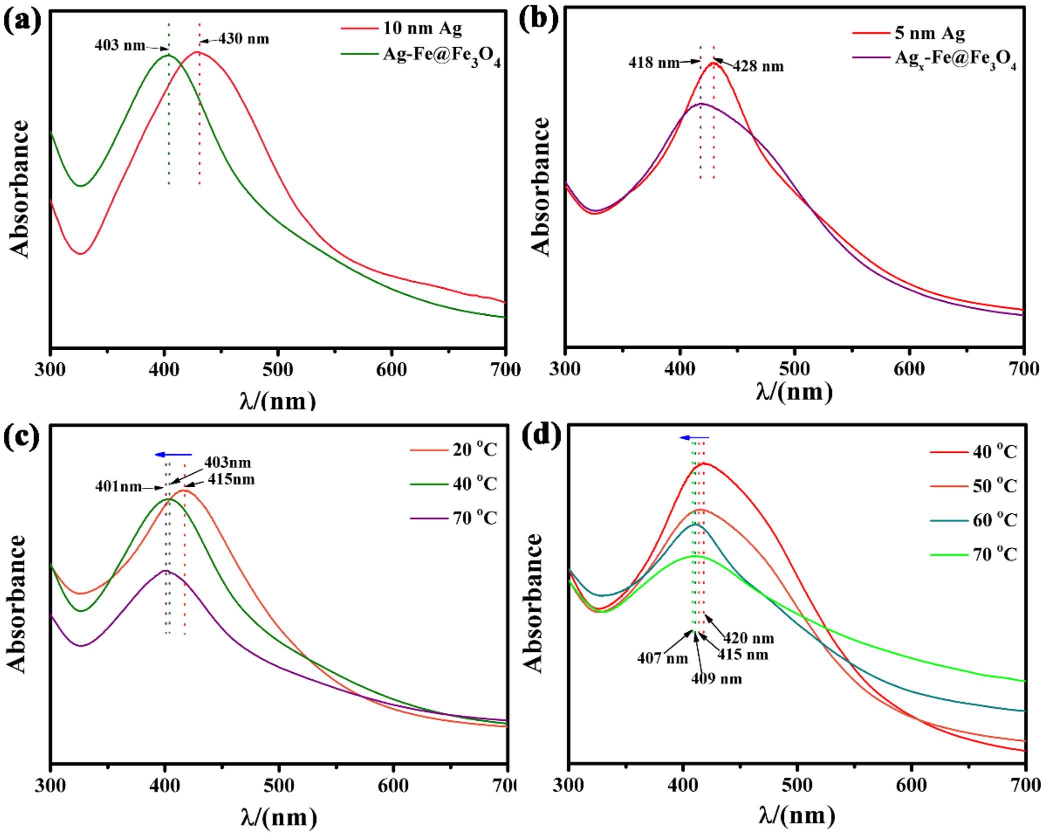

Figure S13. UV-Vis absorption spectra of various nanoparticles. (a) The comparison of Ag NPs (shown in Figure S12b) and JAFHs (shown in Figure 2c) with both 10 nm Ag; (b) the comparison of Ag NPs (shown in Figure S12a) and SAFHs (shown in Figure 3a) with both 5 nm Ag; (c) JAFHs synthesized at different temperature with various Ag sizes as shown in Figure 2b-d; (d) SAFHs synthesized at different temperature with various Ag sizes as shown in Figure 3a,d-f.

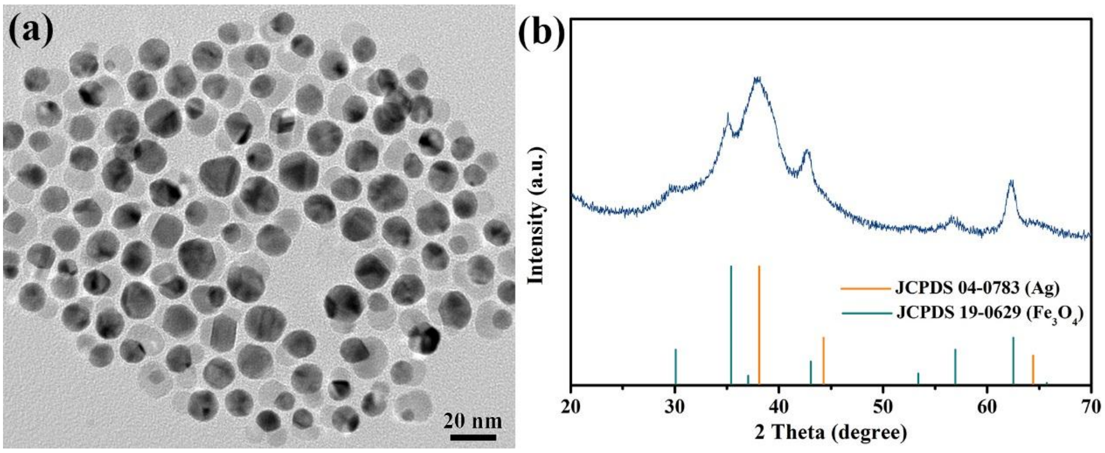

Figure S14. (a) TEM image and (b) XRD patterns of Ag-Fe_3O_4 NPs.

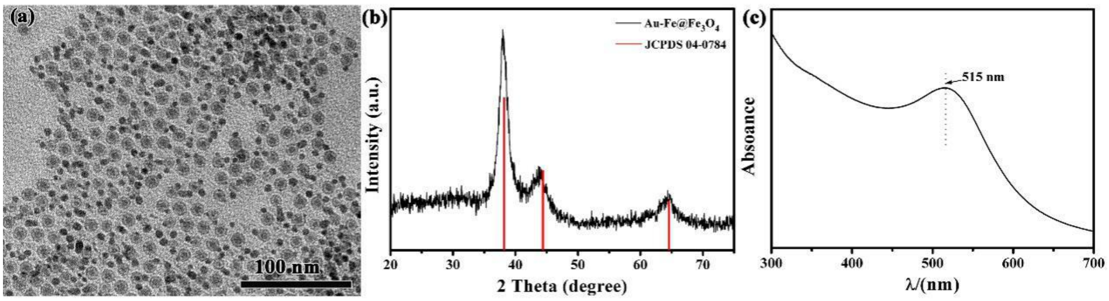

Figure S15. (a) TEM image, (b) XRD patterns and (c) UV-Vis absorption spectra of the as-synthesized Au-Fe@Fe_3O_4 heterostructures consisting of 6 nm Au islands and 13 nm Fe@Fe_3O_4 domains.

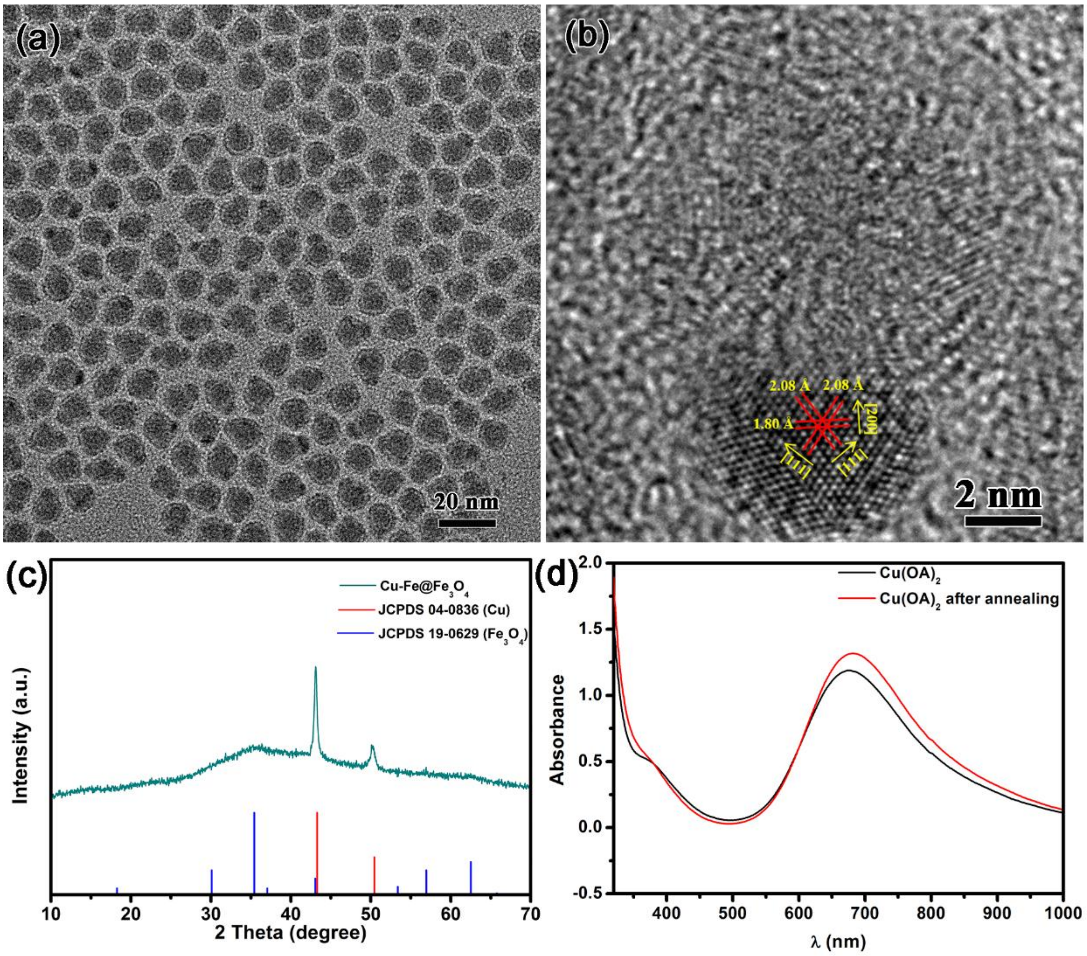

Figure S16. (a) TEM, (b) HRTEM and (c) XRD patterns of Cu-Fe@Fe_3O_4 heterostructures synthesized with self-reduction reaction. (d) UV-Vis absorption spectra of the precursor $Cu(OA)_2$ and its product after being annealing at 120 ^oC.
